# Domain Swapping and Different Oligomeric States for the Complex Between Calmodulin and the Calmodulin-Binding Domain of Calcineurin A

**DOI:** 10.1371/journal.pone.0005402

**Published:** 2009-04-30

**Authors:** Viivi Majava, Petri Kursula

**Affiliations:** Department of Biochemistry, University of Oulu, Oulu, Finland; Naval Research Laboratory, United States of America

## Abstract

**Background:**

Calmodulin (CaM) is a ubiquitously expressed calcium sensor that engages in regulatory interactions with a large number of cellular proteins. Previously, a unique mode of CaM target recognition has been observed in the crystal structure of a complex between CaM and the CaM-binding domain of calcineurin A.

**Methodology/Principal Findings:**

We have solved a high-resolution crystal structure of a complex between CaM and the CaM-binding domain of calcineurin A in a novel crystal form, which shows a dimeric assembly of calmodulin, as observed before in the crystal state. We note that the conformation of CaM in this complex is very similar to that of unliganded CaM, and a detailed analysis revels that the CaM-binding motif in calcineurin A is of a novel ‘1-11’ type. However, using small-angle X-ray scattering (SAXS), we show that the complex is fully monomeric in solution, and a structure of a canonically collapsed CaM-peptide complex can easily be fitted into the SAXS data. This result is also supported by size exclusion chromatography, where the addition of the ligand peptide decreases the apparent size of CaM. In addition, we studied the energetics of binding by isothermal titration calorimetry and found them to closely resemble those observed previously for ligand peptides from CaM-dependent kinases.

**Conclusions/Significance:**

Our results implicate that CaM can also form a complex with the CaM-binding domain of calcineurin in a 1∶1 stoichiometry, in addition to the previously observed 2∶2 arrangement in the crystal state. At the structural level, going from 2∶2 association to two 1∶1 complexes will require domain swapping in CaM, accompanied by the characteristic bending of the central linker helix between the two lobes of CaM.

## Introduction

Calmodulin (CaM) is a calcium-dependent regulatory protein, able to interact with a plethora of target proteins having diverse functions [1]. CaM consists of N- and C-terminal lobes and a central helix. Bending of the central helix is instrumental to conformational changes occurring during target protein recognition. Upon calcium binding to the four EF-hands of CaM, a hydrophobic pocket opens in each of the two lobes of CaM, which then interact with large hydrophobic or aromatic residues from the target sequence.

Originally, the binding of CaM to its target sequence was shown to involve the collapse of CaM around the peptide [2]–[4]; the idea was proven by studies on peptides from CaM-dependent protein kinases (CaMKs) as well as from other proteins [5], [6]. The canonical binding mode is antiparallel, such that the N-terminal half of the peptide interacts with the C-terminal lobe of CaM and *vice versa*. During recent years, a growing number of non-classical modes of binding for CaM towards its target peptides have been detected [1], [7]–[11]. Such modes involve differences in both the conformation of CaM, which may also exist in an extended conformation in a complex, and the stoichiometry of the formed complex, not always being 1∶1.

One example of a non-classical mode of CaM-peptide interaction has been suggested in the case of the calmodulin-binding domain (CBD) of calcineurin A (CnA). Crystal structures have indicated the formation of a 2∶2 complex between CaM and the CnA-CBD. The same kind of complex in the crystal state was observed both when using a CaM-CnA-CBD fusion protein [12] and when cocrystallizing CaM with a synthetic CnA-CBD peptide [13]. Computational studies have, in addition, suggested that the peptides in the complex have a role in stabilizing the 2∶2 association of CaM [14]. Small-angle scattering experiments with longer peptides have, however, been indicative of a 1∶1 complex [15].

Here, we have carried out further in-depth studies on the CaM complex with CnA-CBD. A high-resolution crystal structure of the complex was determined, again showing the 2∶2 complex seen before, and extensive disorder is seen in the hydrophobic pockets of CaM that interact with the peptide. However, other methods provide results that are in clear disagreement with the crystal structures. Size exclusion chromatography indicates that in solution, the complex has a smaller radius than unliganded CaM, excluding the possibility of a 2∶2 complex. Most importantly, synchrotron small-angle X-ray scattering (SAXS) data indicate the presence of a 1∶1 complex, which, in *ab initio* modelling, shows a highly similar conformation to the canonical collapsed mode of CaM-peptide interaction, seen also for peptides from CaMKs. The energetics of binding, determined by calorimetry, also resemble those seen for the canonical mode of binding.

## Results and Discussion

### The high-resolution crystal structure of the complex

**Figure 1:**
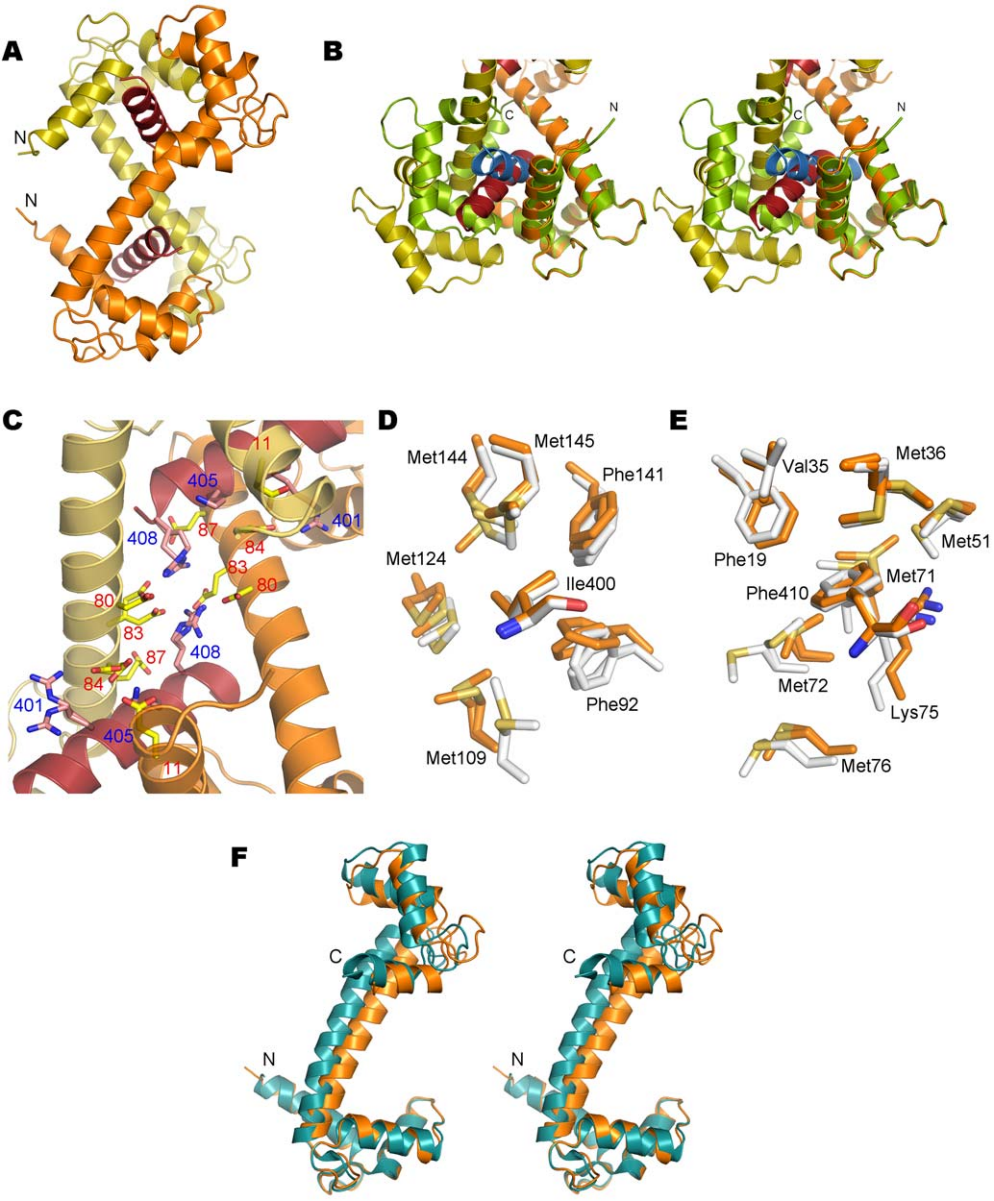
Crystal structure of the CaM-CnA-CBD complex. A. Overall structure. The two peptides are shown in red and the two monomers of CaM in yellow and orange. The N termini of both CaM monomers point to the left. B. Comparison to a classical collapsed structure of a CaM-peptide complex (PDB entry 1WRZ). The superposition was carried out using the N-terminal lobe of CaM (to the right on this view). The colours for the CaM-CnA-CBD complex are as in A, and in the collapsed conformation, CaM is shown in green and the peptide in blue. The location of the hinge, related to the formation of the collapsed conformation, at the middle of the long linker helix is clearly visible. The N and C termini of the collapsed structure are labeled. C. The central part of the X-shaped complex contains a network of salt bridges. Most of the residues involved are mobile in the crystal and have been built in two conformations. The labels for acidic residues from CaM are in red and those for basic residues in the peptide in blue. D. The environment of the N-terminal anchoring residue Ile400 from the peptide in the C-terminal hydrophobic pocket of CaM. Note that all CaM residues forming the pocket are in double conformations. The structure is shown from two different monomers in the asymmetric unit of the structure (white and orange colours). E. The environment of the C-terminal anchoring residue Phe410 from the peptide in the N-terminal hydrophobic pocket of CaM. All 5 methionine residues in the pocket are disordered, and Phe410 is also in a double conformation. Colouring as in D. F. Comparison to the unliganded structure. The stereo view indicates that the structure of the CaM monomer in the complex (orange) is highly similar to that of unliganded CaM (blue).

The complex between CaM and the CnA-CBD peptide was crystallized in a novel space group, comparing to the two earlier crystal structures of this complex [12], [13]. Synchrotron X-ray diffraction data allowed us to solve and refine the structure of the complex at 1.45-Å resolution (Table 1), much higher than previously obtained for the CaM-CnA-CBD complex (between 1.86 and 2.6 Å). It should also be mentioned that only one CaM-peptide complex has been studied at a higher resolution to date: the complex between CaM and a CBD peptide from smooth muscle myosin light chain kinase (PDB entry 2o5g, unpublished). The overall conformation of the CaM-CnA-CBD complex seen in the crystal is the same as in the earlier structures of this complex; most notably, the complex is formed by 2∶2 association, such that CaM forms an X-shaped dimer (Figure 1A). The two peptides in the complex are sandwiched each between the N-terminal lobe from one CaM molecule and the C-terminal lobe from another CaM molecule. The binding mode is somewhat unexpected; while the ‘1-10’ and ‘1-14’ modes are the most common for CaM ligands (the numbering originating from the relative positions in the primary sequence of large hydrophobic residues that bind to the two hydrophobic pockets of CaM), in the case of the CnA-CBD, the binding mode is ‘1-11’. The residues buried in the hydrophobic pockets are, namely, Ile400 and Phe410. The calmodulin target database [16] lists the CnA-CBD as a member of the motif ‘1-5-8-14’, which - based on the current high-resolution structure - is wrong. A comparison to the conformation of a collapsed CaM-peptide complex indicates that the peptide conformation, with respect to the two CaM lobes interacting with it, is also distinct in the CaM-CnA-CBD complex (Figure 1B).

**Table 1:** Crystallographic data collection and structure solution.

Data collection	
Resolution range (Å)	20.0-1.45 (1.49-1.45)
Space group	P2_1_
Unit cell dimensions	
a, b, c (Å)	65.3, 81.3, 71.2
α, β, γ (°)	90.0, 111.3, 90.0
Completeness (%)	98.9 (98.7)
R_sym_ (%)	6.5 (57.5)
Redundancy	4.0 (3.6)
<I/σ(I)>	8.9 (2.7)
Refinement	
Resolution range (Å)	10.0-1.45
R_cryst_ (%)	18.4
R_free_ (%)	21.6
Rmsd bond lengths (Å)	0.020
Rmsd bond angles (°)	1.8
Rmsd B factors of bonded atoms (main chain, side chain) (Å^2^)	2.5, 4.8
Ramachandran plot (%)	
Preferred	97.8
Allowed	2.2
Outliers	0.0

Interestingly, a recent study pointed out that the oxidation of Met406 in the CBD of CnA can lower the affinity towards CaM and prevent calcineurin activation [17]. In the structure, Met406 is close to Phe410, interacting closely with the edge of the N-terminal hydrophobic pocket of CaM, where there are no potential hydrogen bond donors. Thus, the oxidation of Met406 may both affect the conformation of Phe410 and lower the affinity by bringing a hydrophilic oxygen atom into the highly hydrophobic environment.

At the middle of the X-shaped 2∶2 complex, between the two central helices, a network of salt bridges exists, formed by, for example, Arg408 from both peptides and Glu80 and Glu83 from both CaM molecules. The side chains in this salt bridge network are rather disordered, indicating they are probably not forming a rigid network supporting the extended conformation (Figure 1C).

The high resolution of the diffraction data allows the examination of the fine details of peptide recognition in the complex. It is notable that both of the hydrophobic pockets of CaM are relatively disordered, many of the Phe and Met residues being present in a double conformation despite the presence of bound peptide (Figure 1D,E). Also residues in the peptide are rather disordered; these observations bring into question the suggested triggering role of the peptide in the formation of a 2∶2 complex [14]. It should also be noted that the B-factors in the crystal are distributed such that low B-factors are also seen at the exterior of the complex, being involved in crystal contacts, while, as mentioned above, there exists disorder in the peptide and the peptide-binding site. Thus, it is possible that the 2∶2 complex is, at least partially, stabilized by the crystal contacts.

Superposition of the N-terminal half of monomer A from the current and earlier structures indicates flexibility for the central linker, which in all cases is still folded as a long helix. At the other end of the complex, the entire lobe of CaM has moved by approximately 5 Å between some of the structures (not shown). Further superposition analysis indicates also that the intermolecular linkage between the two extended CaM molecules is not tight.

DynDom analysis of domain motions [18] between each CaM chain in the asymmetric unit of our new structure and the previous structures of the complex indicates that the observed conformational change between different liganded forms can mainly be explained by a 15-degree rotation that occurs between residues 80–90, *i.e.* in the C-terminal half of the linker helix (Table S1). In all cases where dynamic domains were identified, there were two domains found. In contrast, comparing the complex to unliganded CaM, there are either 2 or 3 moving domains, depending on which chains are used; the main bending region is at residue 90, at the end of the linker helix, but another one is around residue 60, at the beginning of this helix. Thus, it seems that in the CaM-CnA-CBD complex, CaM is mostly in the unliganded conformation up to residue 90, after which the C-terminal lobe has turned approximately 25° upon peptide binding (Figure 1F). Since this same hinge is observed between the different CnA-CBD complexes, it can be postulated that formation of the complex mainly involves bending at a single site on CaM, and that the bending is not constant in the complex, but significant flexibility exists also in the crystal state. When comparing unliganded CaM to the collapsed conformation, on the other hand, a hinge is seen at the middle of the central helix, at the region containing residues 80–81 (see Figure 1B).

### Energetics of the interaction

**Figure 2:**
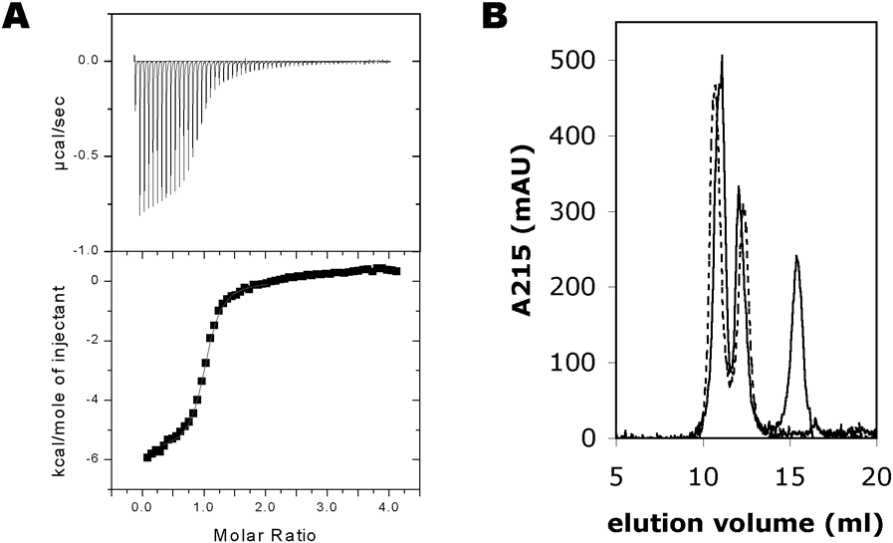
Characterization of the complex by calorimetry and size exclusion chromatography. A. ITC. Top; the original titration curve, bottom; the integrated peak areas were used in curve fitting. B. Size exclusion chromatography of CaM in the absence (dashed line) and presence (solid line) of the CnA-CBD peptide. Note the shift of the first peak (CaM) towards a larger elution volume in the presence of the peptide. The second peak is a form of CaM appearing in CaM samples upon storage, possibly a permanently collapsed form (data not shown). The third peak is the peptide. A(215) is shown in order to visualize the location of the peptide, which does not absorb at 280 nm. The presence of the peptide in the first and third peaks was confirmed by SDS-PAGE (Figure S1).

The binding of the CnA-CBD to CaM was analyzed by isothermal titration calorimetry (ITC), in order to get an accurate estimate of the affinity and thermodynamic parameters of binding (Figure 2A, Table 2). The affinity of CaM towards the peptide is sub-micromolar. The binding stoichiometry from ITC is clearly 1∶1, but it should be noted that a 2∶2 complex is by no means excluded by this result, since such details are indistinguishable in this experiment. The experiment was carried out at two different temperatures, allowing the estimation of the magnitude of the heat capacity. The estimated heat capacity, −0.8 kcal/mol/K, is in the same range as previously observed for collapsed CaM-peptide complexes [19], strongly suggesting a collapsed structure for the complex.

**Table 2:** Calorimetric analysis of the CaM-CnA-CBD interaction.

T	ΔH (kcal/mol)	−TΔS (kcal/mol)	ΔG (kcal/mol)	K_a_ (1/M)	K_d_ (µM)
+30°C	−1.8	−7.3	−9.1	3.5×10^6^	0.3
+35°C	−5.7	−3.0	−8.7	1.4×10^6^	0.7

### Size exclusion chromatography

The solution properties of CaM alone and in complex with the CnA-CBD were initially analyzed with size exclusion chromatography. Unliganded CaM, due to its elongated dumbbell shape, runs at an apparent molecular weight of 25–30 kDa. If the complex has a structure similar to that seen in the crystal structure, one would expect it to elute from the column faster than CaM alone; the 2∶2 arrangement is made of two elongated CaM molecules, which sandwich two CnA-CBD peptides between them. Thus, the expected hydrodynamic radius for the 2∶2 complex would be significantly higher than for CaM. However, the complex actually elutes from gel filtration later than CaM (Figure 2B, Figure S1), suggesting a smaller hydrodynamic radius; similar behaviour was observed earlier with a peptide from myelin basic protein [9]. This implies both that the complex is not 2∶2 but 1∶1 in solution, and that CaM undergoes a conformational change in the presence of the peptide that decreases its hydrodynamic radius. The latter options both are compatible with the classical collapse of CaM around a target peptide.

When using a fusion protein of CaM and the CnA-CBD, Ye and coworkers [12] observed a very small amount of dimeric complex under gel filtration, while it can be estimated visually from their result that >99% was present as a monomer in the experiment; the scale on the shown chromatogram was actually logarithmic. Thus, at least in the absence of interactions stemming from other parts of CnA than the CBD segment, the CaM-CnA-CBD complex is quantitatively monomeric in solution.

### Solution structure determination using small-angle X-ray scattering

**Figure 3:**
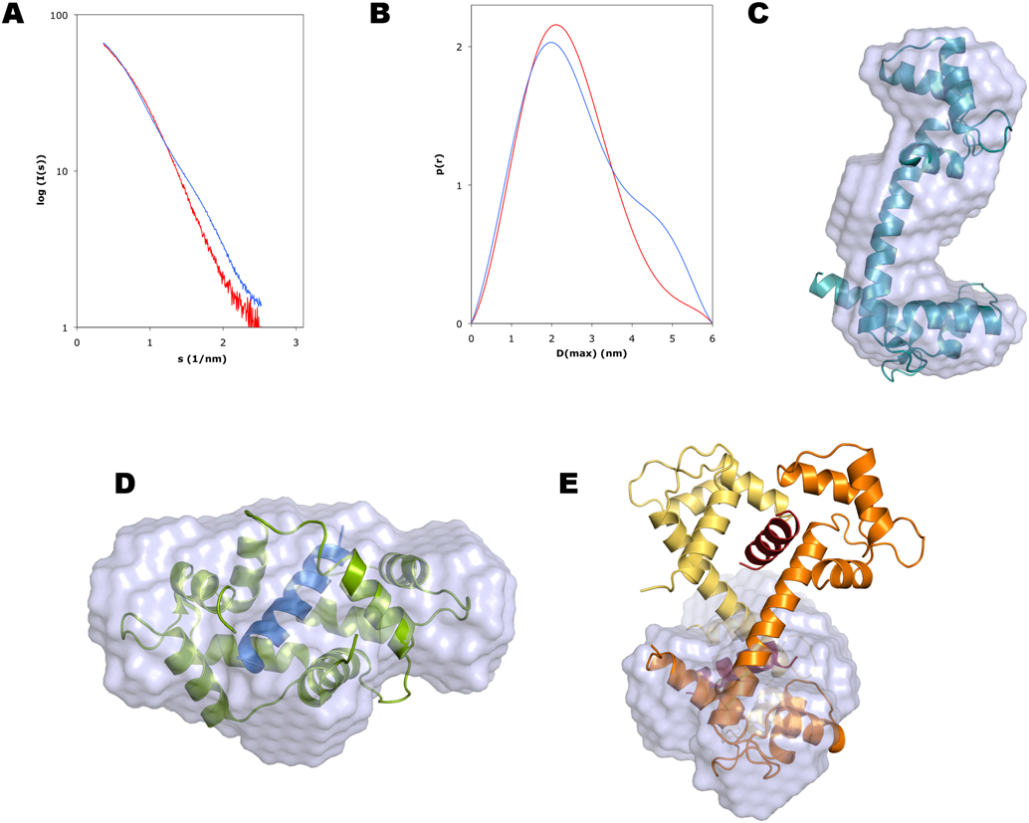
Small-angle X-ray scattering analysis of the solution properties of the complex. A. Scattering curve. Superposed are the curves for CaM (blue) and the complex (red). B. Distance distribution function. Colouring as in A. Note the disappearance of the shoulder around 4.5 nm in the presence of the peptide. The shoulder is characteristic of the dumbbell-shaped conformation of unliganded CaM, while the distance distribution for the complex indicates a more globular shape. C. *Ab initio* model of CaM, with the crystal structure of unliganded CaM superimposed. D. Superposition of the *ab initio* complex solution structure with the crystal structure of a canonical CaM-peptide complex (PDB code 1WRZ). E. Superposition of the SAXS structure with half of the crystal structure of the complex. Colouring as in Figure 1A.

The solution properties of the complex were studied in more detail using synchrotron small-angle X-ray scattering (Figure 3). Scattering curves were measured for both CaM and the complex. The SAXS data clearly indicate that the complex is monomeric (1∶1 stoichiometry), as observed by comparing the values describing the size and shape of the particle to those obtained for the CaM sample, as well as to the theoretical values calculated from different crystal structures using the program CRYSOL (Table 3).

**Table 3:** Parameters from SAXS and comparison to theoretical values.

Sample	R_g_ (nm)	MW (kDa)	D_max_ (nm)	Displaced volume (nm^3^)
CaM (SAXS)	2.05	21.3	6	29.5
CaM+CnA-CBD (SAXS)	1.85	19.6	5.5	29.9
CaM+CnA-CBD (crystal)	2.25	38.4	7.7	47.4
CaM+CnA-CBD (half) (crystal)	1.72	18.4	5.5	22.6
1WRZ (crystal structure of a collapsed complex)	1.70	20.7	5.4	23.8
1UP5 (crystal structure of unliganded CaM)	2.29	16.7	7.0	20.4

For the SAXS samples, the molecular weight was estimated by comparing the forward scattering to that of bovine serum albumin, and the displaced volume is that of the average *ab initio* model from DAMMIN. For the crystal structures, all the values have been calculated using CRYSOL.

**Figure 4:**
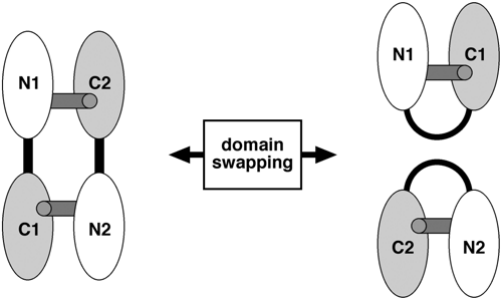
Schematic view of the transition between the two observed complexes. Domain swapping is involved in the transition between the 1∶1 and 2∶2 complex, as seen in the solution and crystal structures, respectively. The peptides are represented by gray sticks, and the central linker helix is shown in black. The N- and C-terminal lobes of both CaM molecules are also labeled.

*Ab initio* model building was also carried out for both CaM and the complex in DAMMIN (Figure 3C,D). For the complex, the modeled structure is clearly reminiscent of the collapsed mode of CaM-peptide interaction, and a structure of a CaM-CaMK peptide complex can easily be fitted into the model (Figure 3D). Furthermore, the modeled structure apparently represents one-half of the structure observed in the crystal structure, indicating the presence of a collapsed 1∶1 complex (Figure 3E). Between such a complex and the crystal structure, the transformation must involve domain swapping of the lobes of CaM (Figure 4). In the 2∶2 complex, N- and C-terminal lobes from two different CaM molecules interact with the same peptide, while in the case of the 1∶1 complex, the interaction between the peptide and the lobes is similar, but both CaM lobes come from the same molecule, and the central helix must be bent.

### Concluding remarks

We have shown here that while CaM forms a 2∶2 complex with CnA-CBD in the crystal state, the complex is of 1∶1 stoichiometry, with a collapsed conformation, in solution. The results further highlight the plasticity of CaM in target recognition; *via* domain swapping, CaM is able to bind to CnA in a 1∶1 or 2∶2 ratio. Our data provide for the first time accurate 3D structural data on the 1∶1 complex formed between CaM and the CnA-CBD in solution, and unambiguously show that CaM adopts a classical collapsed conformation upon CnA-CBD peptide binding. The observed small hinge movement between the crystal structure and unliganded CaM may represent an early stage of CaM collapse around the CnA peptide, as seen in solution studies. In addition, a novel mode of CaM target recognition has been recognized based on our high-resolution crystallographic data, being based on a ‘1-11’ motif, not predictable from the sequence of the CnA-CBD.

## Materials and Methods

### Protein and peptide preparation

Human (vertebrate) calmodulin was expressed in *E. coli* using the pETCM vector as earlier described [20], [21]. Briefly, CaM was purified using calcium-dependent hydrophobic interaction chromatography on phenyl sepharose, concentrated using centrifugal ultrafiltration, and dialyzed into 10 mM HEPES (pH 7.5), 100 mM NaCl, 10 mM CaCl_2_. The peptide corresponding to the CaM binding domain of CnA (residues 395–411, sequence VIRNKIRAIGKMARVFS) was purchased from GL Biochem (Shanghai, China), dissolved in water, and dialyzed into the buffer given above.

### Isothermal titration calorimetry

Calorimetry was performed using the Microcal VP-ITC apparatus. CaM (0.1 mM) was placed in the cell, and the CnA-CBD peptide (1 mM) in the syringe, and injections of 5 µl were performed. All dilutions were done with the used dialysis buffer. The experiment was carried out in an identical fashion at temperatures of +35 and +30°C, and the data were analyzed using Microcal Origin. After correcting for the heat of dilution, thermodynamic parameters for the interaction were obtained from curve fitting of the titration data.

### Size exclusion chromatography

The mobility of CaM in the presence and absence of the CnA-CBD was analyzed by size exclusion chromatography, as previously described for a peptide from the myelin basic protein [9]. Briefly, CaM (200 µl at 176 µM) was run through a Superdex 75 HR 10/30 column, in the presence and absence of excess (625 µM) CnA-CBD peptide, using 50 mM HEPES (pH 7.5), 150 mM NaCl, 4 mM CaCl_2_ as the running buffer. Protein elution was followed by measuring absorbance at 215 and 280 nm, and protein-containing fractions were analyzed by SDS-PAGE.

### Crystallization and data collection

The CaM-CnA-CBD complex (10 mg/ml) was crystallized at room temperature using the hanging drop vapour diffusion method, with a well solution consisting of 30% PEG 400, 200 mM KCl, and 100 mM Tris (pH 8.5). The drop contained 1 µl of protein solution and 1 µl of well solution, and equilibration was carried out over 500 µl of well solution. Prior to data collection, the crystals were cryocooled in liquid nitrogen. Diffraction data were collected at 100 K on the Cassiopeia beamline I911-3 at MAX-Lab, Lund, Sweden [22]. Data were processed with XDS [23] and XDSi [24]. 5% of all reflections were set aside for calculating the free R factor.

### Crystal structure solution and refinement

The structure was solved by molecular replacement using Molrep [25], and the structure of the CaM-CnA-CBD fusion protein was used as a model. Refinement was carried out in Refmac [26], making use of TLS parameters [27], [28], and model building in Coot [29]. Water molecules were picked both manually and using the relevant routines in Coot. The coordinates and structure factors have been deposited at the Protein Data Bank with the accession code 2W73.

### Small-angle X-ray scattering

For SAXS, CaM and the CnA-CBD were dialyzed into a buffer containing 50 mM HEPES (pH 7.5), 150 mM NaCl, and 10 mM CaCl_2_. SAXS data for CaM in the presence and absence of the peptide, at concentrations ranging between 1 and 10 mg/ml, were measured on the EMBL Hamburg/DESY beamline X33, and the corresponding buffer was always used for a blank experiment. Programs from the ATSAS package [30] were used for data analysis, essentially as earlier described [9], [31]. The measured data were further processed using PRIMUS [32], where the scattering by the buffer was subtracted and the scattering normalized to a concentration of 1 mg/ml. The molecular weight was estimated by comparing the forward scattering I(0) to that of a standard solution of bovine serum albumin. The distance distributions were obtained using GNOM [33], and further used for *ab initio* modelling in DAMMIN [34]. An averaged model was generated from several runs using programs of the DAMAVER package [35], and the SAXS model and the crystal structures were superimposed with SUPCOMB [36]. The calculation of the theoretical solution scattering of different crystal structures was carried out using CRYSOL [37].

## Supporting Information

Figure S1 SDS-PAGE analysis of the gel filtration fractions.
(0.22 MB DOC)

Table S1 DynDom analysis of domain motions between different CaM chains
(0.04 MB DOC)
