# Supplementary material for: Domain Swapping and Different Oligomeric States for the Complex Between Calmodulin and the Calmodulin-Binding Domain of Calcineurin A
Source: PLoS One. 2009 Apr 30;4(4):e5402. doi: 10.1371/journal.pone.0005402 (PMC2671406; doi:10.1371/journal.pone.0005402)

***Figure S1. SDS-PAGE analysis of the gel filtration fractions.***

The samples are as follows: 1, the loaded sample; 2-9, fractions 10-17, respectively. The first eluted peak is in fractions 10-11, the second peak in fraction 12, and the third in fractions 15-16. Although the peptide stains weakly, it is visible in the first and third peaks.


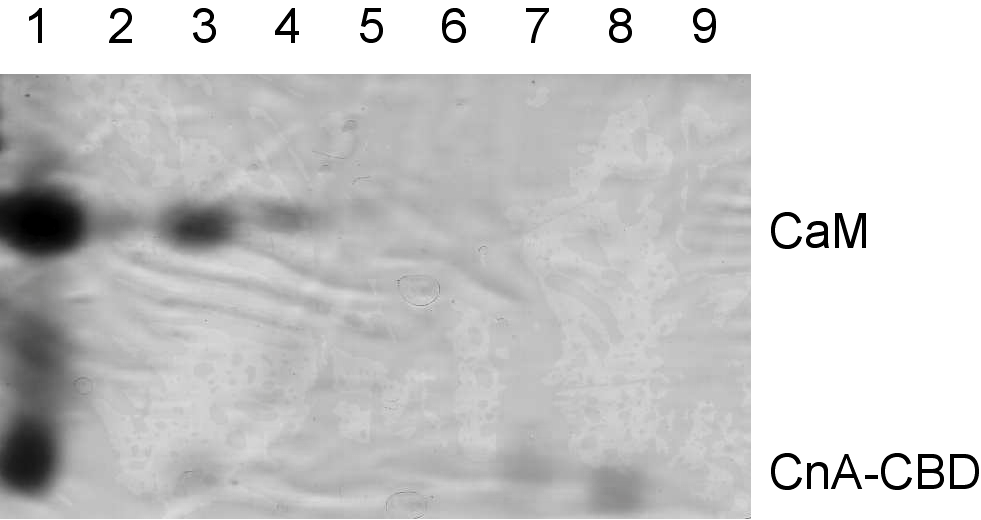

Supplement: Figure S1 — SDS-PAGE analysis of the gel filtration fractions. (0.22 MB DOC) [file pone.0005402.s001.doc]
