# Supplementary material for: Domain Swapping and Different Oligomeric States for the Complex Between Calmodulin and the Calmodulin-Binding Domain of Calcineurin A
Source: PLoS One. 2009 Apr 30;4(4):e5402. doi: 10.1371/journal.pone.0005402 (PMC2671406; doi:10.1371/journal.pone.0005402)
Supplement: Table S1 — DynDom analysis of domain motions between different CaM chains (0.04 MB DOC) [file pone.0005402.s002.doc]

Table S1. DynDom analysis of domain motions between different CaM chains.

| Chain | Chain in the current structure | | | |
| --- | --- | --- | --- | --- |
|  | A | B | E | F |
| Current A | - | 7-85,138-142.0.55 Å  86-137, 0.66 Å  85-86,137-138  8.3 °, 7.8 %, 0.93 Å | 1.04 Å, not found | 7-32,56-145, 0.86 Å  33-53, 0.27 Å  32-33,53-58  12.0 °, 60.3 %, 1.04 Å |
| Current B |  | - | 5-77, 0.44 Å  78-148, 0.88 Å  77-78  9.8 °, 34.6 %, 1.20 Å | 0.89 Å, not found |
| Current E |  |  | - | 5-82, 0.36 Å  83-144, 0.88 Å  82-83  6.7 °, 15.9 %, 0.97 Å |
| Current F |  |  |  | - |
| 2R28 A | 7-90,135-143,0.81Å  91-95,100-134, 0.84 Å  90-91,95-100,134-135  11.7 °, 74.1 %, 1.27 Å | 7-91,95-99,128-143, 0.79 Å  92-94, 100-127, 0.85 Å  91-92,94-95,99-100,127-128  14.3 °, 93.1 %, 1.25 Å | 7-81, 0.41 Å  82-144, 1.3 Å  78-82  15.6 °, 76.0 %, 1.71 Å | 7-89, 0.54 Å  90-145, 0.65 Å  85-90  14.1 °, 99.1 %, 1.37 Å |
| 2R28 B | 7-32,54-144. 1.18 Å  33-53, 0.3 Å  32-33, 53-54  12.7 °, 72.2 %, 1.28 Å | 1.19 Å, not found | 11-85, 0.57 Å  86-144, 1.34 Å  85-86  12.5 °, 69.4 %, 1.45 Å | 1.25 Å, not found |
| 2F2O A | 8-89,97-99,135-144,0.78 Å  90-96,100-134, 0.82 Å  89-90,96-97,99-100,134-135  11.9 °, 73.7 %, 1.27 Å | 7-91, 95-99, 127-143, 0.78 Å  92-94, 100-126, 0.87 Å  91-92, 94-100. 125-127  14.6 °, 93.8 %, 1.26 Å | 7-81, 0.46 Å  82-144, 1.25 Å  79-82  16.4 °, 75.6 %, 1.73 Å | 7-86, 0.49 Å  87-145, 0.63 Å  81-87  15.0 °, 98.4 %, 1.42 Å |
| 2F2O B | 6-69, 0.66 Å  70-145, 0.74 Å  69-70  7.9 °, 6.2, 1.01 Å | 0.74 Å, not found | 16-81, 0.44 Å  82-144, 0.77 Å  81-82  11.5 °, 30.0 %, 1.16 Å | 16-84, 0.41 Å  85-145, 0.7 Å  84-85  8.3 °, 84-85 %, 0.90 Å |

Where domains were found, the first line indicates the residues and rmsd in domain 1, the second line the residues and rmsd in domain 2, the third line the bending region, and the fourth line the rotation angle, percentage of closing motion, and the overall rmsd. If no domains were found, only the overall rmsd is shown. 2F2O refers to the structure of the CaM-CnA-CBD fusion protein, and 2R28 to the structure of the corresponding CaM-peptide complex determined earlier.
